# The pulmonary-vascular-stump filling defect on CT post lung tumor resection: a predictor of cancer progression

**DOI:** 10.1186/s40644-024-00739-y

**Published:** 2024-07-16

**Authors:** Lei Ni, Qihui Wang, Yilong Wang, Yaqi Du, Zhenggang Sun, Guoguang Fan, Ce Li, Guan Wang

**Affiliations:** 1 Department of Radiology, The First Hospital of China Medical University, No.155, North Nanjing Street, Shenyang, 110001 Liaoning China; 2 Department of Clinical Laboratory, The First Hospital of China Medical University, Shenyang, China; 3 Department of Radiotherapy department, The First Hospital of China Medical University, Shenyang, China; 4 Department of Medical Oncology, The First Hospital of China Medical University, No.155, North Nanjing Street, Shenyang, 110001 Liaoning China

**Keywords:** Pulmonary vascular stump filling defect, Lung cancer, Cancer progression, Computed tomography

## Abstract

**Background:**

To explore the pulmonary-vascular-stump filling-defect on CT and investigate its association with cancer progression.

**Methods:**

Records in our institutional database from 2018 to 2022 were retrospectively analyzed to identify filling-defects in the pulmonary-vascular-stump after lung cancer resection and collect imaging and clinical data of patients.

**Results:**

Among the 1714 patients analyzed, 95 cases of filling-defects in the vascular stump after lung cancer resection were identified. After excluding lost-to-follow-up cases, a total of 77 cases were included in the final study. Morphologically, the filling-defects were dichotomized as 46 convex-shape and 31 concave-shape cases. Concave defects exhibited a higher incidence of increase compared to convex defects (51.7% v. 9.4%, *P* = 0.001). Among 61 filling defects in the pulmonary arterial stump, four (6.5%) increasing concave defects showed the nuclide concentration on PET and extravascular extension. The progression-free survival (PFS) time differed significantly among the concave, convex, and non-filling-defect groups (log-rank *P* < 0.0001), with concave defects having the shortest survival time. Multivariate Cox proportional hazards analysis indicated that the shape of filling-defects independently predicted PFS in early onset on CT (HR: 0.46; 95% CI: 0.39–1.99; *P* = 0.04). In follow-ups, the growth of filling-effects was an independent predictor of PFS (HR: 0.26; 95% CI: 0.11–0.65; *P* = 0.004).

**Conclusions:**

Certain filling-defects in the pulmonary-arterial-stump post lung tumor resection exhibit malignant growth. In the early onset of filling-defects on CT, the concave-shape independently predicted cancer-progression, while during the subsequent follow-up, the growth of filling-defects could be used independently to forecast cancer-progression.

## Background

With the increasing number of lung cancer surgeries, the detection rate of filling defects in the postoperative pulmonary vascular stump has risen, with reported prevalence ranging from 1.9 to 12.4% [1–7]. According to limited study reports, these defects are often considered as intravascular clots with a benign natural history [1, 5, 6]. In fact, we cannot dismiss the potential for malignancy in these defects, which may indicate vascular stump recurrence in cancer patients. However, as of now, there is a lack of longitudinal observation and verification regarding the association between intravascular filling defects and vascular stump recurrence.

Furthermore, the close association between cancer and coagulation has been emphasised since Armand Trousseau initially described the association between blood clots and cancer in 1865 [8]. Cancer cells can directly activate the coagulation system or do so indirectly through various factors and cytokines. Consequently, the activated coagulation system plays a role in tumor development and the formation of metastasis [9]. Despite this, current research predominantly concentrates on the risk factors for the formation of filling defects and its own outcomes [1, 2, 4, 7], with a lack of investigation into follow-ups and its correlation with cancer prognosis. Thus, we designed this study to characterize the filling defect in the postoperative pulmonary vascular stump and evaluate its association with disease progression following lung cancer resection.

## Methods

This retrospective study was approved by our institutional review board, which waived informed consent. The study enrolled all patients who underwent lung cancer resection and had chest CT enhancement scan or pulmonary artery CTA at our institution between 2018 and 2022. Patient imaging data were obtained from the radiology department database, and clinical data were retrieved from our electronic medical record system. Postoperative chest CT scans were retrospectively reviewed, with inclusion criteria: (a) confirmed lung cancer by postoperative pathology, (b) underwent lobectomy or pulmonary resection surgery, (c) postoperative follow-up with pulmonary CT angiography (CTA) or enhanced lung CT. Patients without hospitalization information or subsequent CT imaging examinations were excluded. The imaging examinations were analyzed and confirmed by two cardiothoracic radiologists with ten years of experience. The filling-defect group was defined as CT scans showing isolated filling defects of lung vessels adhered to the surgical suture lines without extraluminal extension.

**Fig. 1:**
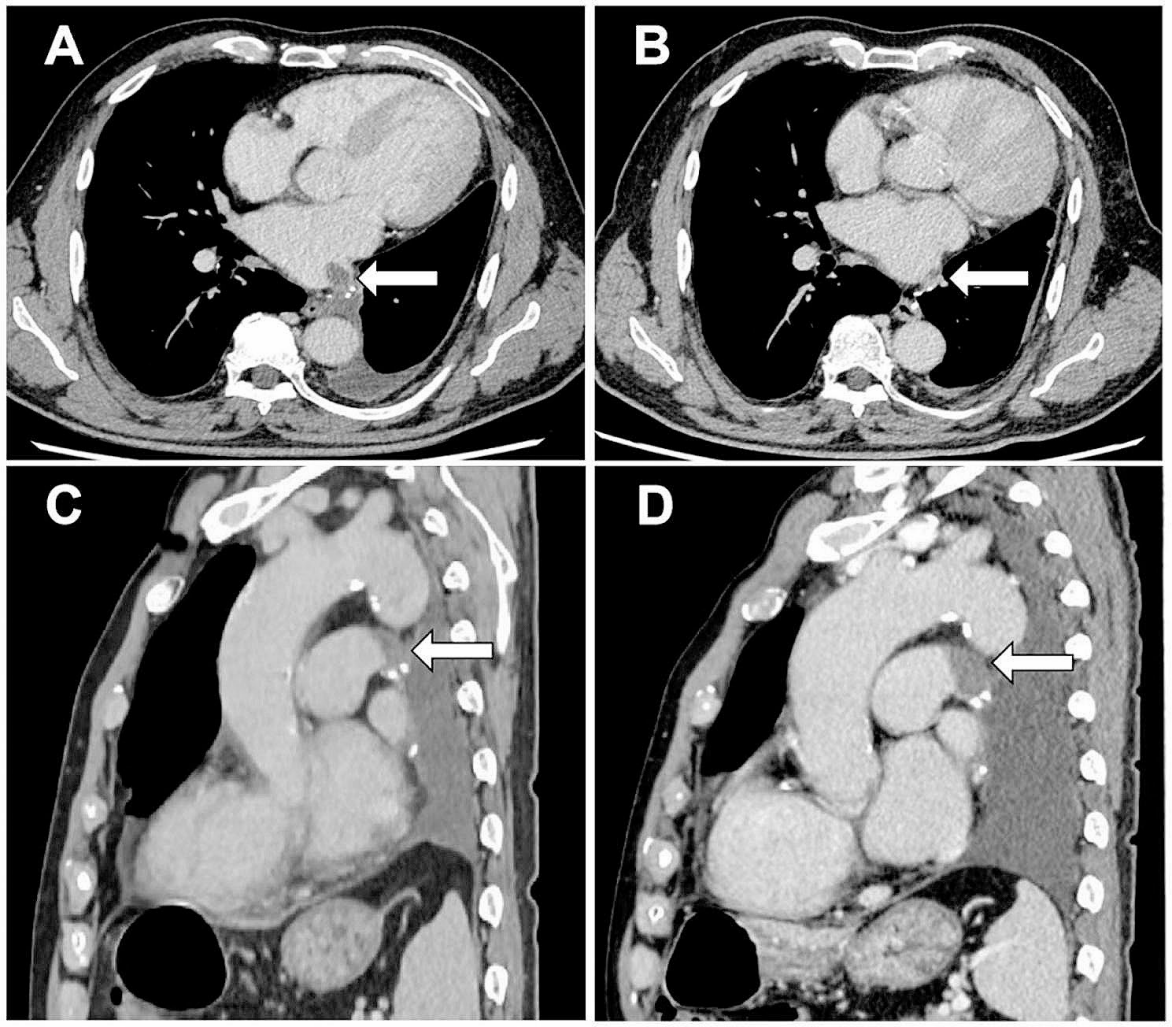
Evolution of pulmonary vascular stump filling defects over follow-up. (**A**) Initial detection of a convex-shaped filling defect in the left inferior pulmonary venous stump. (**B**) Subsequent follow-up CT scan 232 days later revealing partial spontaneous resolution of the filling defect (white arrow indicates the vascular stump). (**C**) Identification of a concave-shaped filling defect in the left main pulmonary arterial stump. (**D**) Progressive increase in the filling defect observed 169 days post-onset (white arrow indicates the vascular stump)

Morphologically, descriptive terms “Convex” and “Concave” were utilized to characterize the filling defects during this study. Convex-shaped filling defect presents as a smooth nodular filling defect with a sharp margin that forms a convex margin with acute angles to the pulmonary vessel wall. Concave-shaped filling defect is characterized by a concave margin (forms a cap at the tip of the stump), the filling defect adhered to one or both ends of the suture line, with extension along the vessel wall and form a blunt angle [1] (Fig. 1). Patients in our study could be divided into three groups: convex-shaped filling-defect group, concave-shaped filling-defect group, and no filling-defect group. The no filling-defect group was matched to the filling-defect groups based on admission time and randomly sampled in a 1:1 ratio.

Imaging data encompassed the vascular stump length, location, time, size, morphology, and attenuation of filling defects. Filling defects change during follow-ups. The vascular stump length was measured from 3D CT images, capturing the maximum measurement along the longitudinal axis of the residual end. As the exact time of initial filling defect detection could not be precisely determined, we recorded the early onset time, that is when the filling defect was initially detected during routine follow-up chest CT enhancement. The first follow-up CT after lung cancer surgery in our institution is one month, then follow-up CT scans are performed every three months if the result is normal. Attenuation was measured by avoiding artefacts, taking three measurements at the centre of the filling defect, and using the median value.

Clinical data, including surgical, clinical, and tumor variables, were collected from the electronic medical record system. These data encompassed surgical date, location of lung resection, histopathology, based on international classification, preoperative tumor staging, tumor progression and death during follow-ups. Tumor progression is considered if at least one of the following criteria is met: the tumor increases in length by ≥ 20%, new lesions appear, or there is unmeasurable progression of malignant lesions. Comorbidities such as hypertension, diabetes, coronary heart disease, smoking, obesity (body mass index ≥ 30 kg/m²), D-Dimer, Fibrinogen Degradation Products abnormal and anticoagulant use after the defection of filling defects were also recorded from the electronic medical record system at the time of patient admission.

Progression-free survival (PFS) serves as a valuable outcome measure in evaluating patients. It can be defined as the time from the detection of filling defects until the patient undergoes endpoint event, endpoint event refer to patient undergoes tumor progression or all-cause death.

Data analysis was conducted using IBM SPSS Statistics (Version 22). Patients were categorized into three groups: no filling defect, convex-shaped filling defect, and concave-shaped filling defect. Continuous variables were presented as mean ± standard deviation, while categorical variables were expressed as frequencies and percentages. The Kolmogorov-Smirnov test assessed data normality. For comparisons of categorical variables, the chi-square test or Fisher’s exact test was used. Student’s t-test compared means or medians of normally distributed data, whereas the non-parametric Mann-Whitney U-test analysed non-normally distributed data. A *P* value less than 0.05 indicated statistical significance.

To evaluate the correlation between the filling defect formation and tumor progression, the Kaplan-Meier test was used for survival analysis, with pairwise comparison of the three study groups. Finally, we employed univariate and multivariate Cox proportional hazards models to assess the effect of the filling defect formation on tumor progression and estimate hazard ratios (HRs) and 95% confidence intervals (CIs). The risk factors for tumor progression after a filling defect in the pulmonary vascular stump were examined in nine pre-defined subgroups (gender, age, histological types, cancer stage at surgery, surgical intervention, location, shape, attenuation (Hounsfield unit), and findings at follow-ups) by incorporating the subgroup variables into the Cox model.

## Results

A total of 1714 patients who underwent lung cancer resection and chest CT enhancement scan or pulmonary artery CTA from January 2018 to August 2022 were screened. Among them, 95 (5.5%) cases of postoperative pulmonary-vascular-stump filling defects were detected, and 18 patients were lost to follow-up due to the lack of hospitalization information or subsequent examinations. Ultimately, 77 patients were included, comprising 61 cases of pulmonary artery stump filling defects and 16 cases of pulmonary vein stump filling defects. The convex-shaped filling-defect group had 46 cases, and the concave-shaped thrombus group had 31 cases. The no filling-defect group of 80 subjects was randomly selected. Table 1 displays the clinical characteristics and baseline comorbidities.

**Table 1:** Clinical characteristics and baseline comorbidities summary

	Pulmonary vascular stump filling defects			*P*
	No (*N* = 80)	Convex (*N* = 46)	Concave (*N* = 31)	
Gender:				0.026
Male	49(61.3%)^#^	38(82.6%)	24(77.4%)	
Female	31(38.8%)	8(17.4%)	7(22.6%)	
Age, years:	62.6 ± 8.56	61.8 ± 7.11	63.7 ± 7.14	0.609
Histological types:				0.501
Large cell	2(2.50%)	2(4.35%)	1(3.23%)	
Squamous cell	21(26.2%)	18(39.1%)	9(29.0%)	
Adenocarcinoma	53(66.2%)	22(47.8%)	18(58.1%)	
Small cell	4(5.00%)	4(8.70%)	3(9.68%)	
Stage of cancer at surgery:				0.270
Stage I/II	61(76.3%)	29(63.0%)	21(67.7%)	
Stage III/IV	19(23.7%)	17(37.0%)	10(32.3%)	
Surgical intervention:				0.322
Right side	50(62.5%)	23(50.0%)	16(51.6%)	
Left side	30(37.5%)	23(50.0%)	15(48.4%)	
Length of vascular stump (cm)	0.61 ± 0.73^&^	1.33 ± 0.81	1.60 ± 0.89	< 0.001
Tumor progression	22(27.5%)	12(26.1%)	18(58.1%)*	0.003
Diabetes mellitus:	9(11.2%)	6(13.0%)	5(16.1%)	0.785
Arterial hypertension:	15(18.8%)	7(15.2%)	10(11.2%)	0.167
Coronary disease:	6(7.50%)	7(15.2%)	3(3.22%)	0.392
Smoking:	12(15.0%)	9(19.6%)	7(22.6%)	0.604
BMI (BMI ≥ 25):	4(5.00%)	5(10.9%)	1(11.2%)	0.419
Previous VTED:	7(8.75%)	7(15.2%)	4(3.22%)	0.505
Atrial fibrillation:	3(3.75%)	1(2.17%)	1(3.22%)	1.000
Fibrinogen Degrdtion Product abnormal	3(3.75%)	3(6.52%)	4(12.9%)	0.245
D-Dimer>500ug/l	2(4.82%)	3(6.52%)	3(9.68%)	0.270

^#^ Compared to the convex-filling defect group, the difference is statistically significant *P* = 0.013, ^#^ compared to concave-filling defects, the difference is not statistically significant *P* = 0.107

^&^ Compared to the filling defect group, the difference is statistically significant *P*<0.001, convex-filling defects compared to concave-filling defects, the difference is not statistically significant *P* = 0.145

* Compared to the no filling defect group, the difference is statistically significant *P* = 0.002, * Compared to convex-filling defects, the difference is statistically significant *P* = 0.005

The concave-shaped filling-defect group showed a higher number of tumor progressions compared to the no filling-defect group and the convex-shaped filling-defect group, with statistically significant differences (*P* = 0.002, *P* = 0.005).

### CT features and imaging follow-ups of filling defects

The onset time was significantly shorter for convex-shaped filling defects (median: 49 days, IQR: 34–116 days) compared to concave-shaped filling defects (median: 884 days, IQR: 148–2203 days) (*P* < 0.001). The long axis of concave-shaped filling defects was larger than that of convex-shaped filling defects (*P* = 0.010). However, there were no statistically significant differences in the attenuation and stump length between the two types of filling defects.

**Fig. 2:**
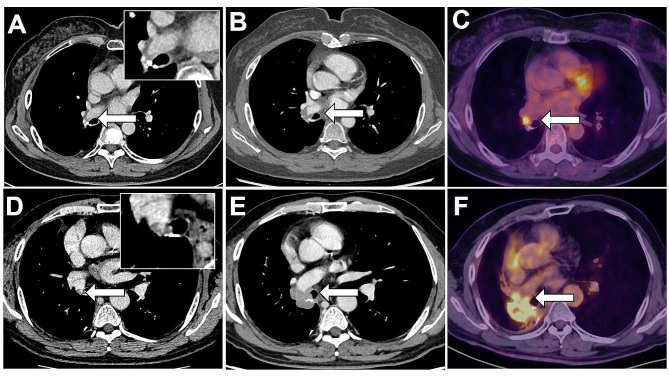
The malignant progression of concave-shaped pulmonary arterial stump filling defects. (**A**) A 64-year-old female lung cancer patient exhibiting a concave-shaped filling defect in the pulmonary arterial stump on CT 134 days post-lobectomy. (**B**) Progressive increase in the filling defect observed 65 days post-onset. (**C**) Corresponding PET-CT image of highlighting fluorodeoxyglucose concentration in the filling defect. (**D**) A 63-year-old male lung cancer patient displaying a oncave-shaped filling defect in the pulmonary arterial stump on CT 222 days post-lobectomy. (**E**) Following anticoagulant therapy with Rivaroxaban, the follow-up CT scan revealed an increased filling defect and the recurrence of the vascular stump 180 days post-onset. (**F**) Corresponding PET-CT image of showing fluorodeoxyglucose concentration in both the recurrent neoplasm and the filling defect

Out of the 77 filling defects, 54 exhibited stable or regression during follow-ups, while 23 increased in size. All 16 filling defects of the pulmonary venous stump, including three that underwent anticoagulant therapy, showed regression during the follow-up, with no occurrences of secondary peripheral embolism. Among 61 filling defects of the pulmonary arterial stump, four (6.5%) increasing concave-shaped filling defects showed nuclide concentration on PET, which developed into extravascular extension from intra-artery filling defects during CT follow-ups(Fig. 2). The CT findings of these 4 cases of cancer recurrence showed that the vascular stump concave filling defects spread along the wall of the tube with irregular edges, the filling defects in the lung stump were small, and CT scans initially showed a pseudo-shadow around the lesion, making it difficult to assess the lesion’s blood supply by CT contrast enhancement. The follow-up CT showed that the filling defects may be intraluminal expansion, or may spread outside the lumen to form a soft tissue mass, enhanced scan showed obvious enhancement.

Among the filling defects without anticoagulant treatment (*n* = 61), 43 (70.4%) showed stability or regression. Specifically, 29 out of 32 (90.6%) convex-shaped filling defects remained stable or regressed, whereas, only 14 out of 29 (48.3%) concave-shaped filling defects remained stable or regressed. There is a significant difference in the rate of spontaneous regression between convex-shaped and concave-shaped filling defects (*P* = 0.001)(Table 2), with convex-shaped filling defects more likely to regress. There were 26 cases of completely resolved filling defects, including 20 cases of convex and six cases of concave-shaped filling defects. The time taken for complete resolution showed no significant difference between the two groups (*P* = 0.694)(Table 2), being 223 days (IQR: 108–456 days) for convex-shaped filling defects and 204 days (IQR: 29–992 days) for concave-shaped filling defects, respectively.

**Table 2:** CT characteristics and Follow-Up of Pulmonary-Vascular-Stump Filling defects Post-lung Cancer Resection

	Convex (*N* = 46)	Concave (*N* = 31)	*P*
Time of first appearance of filling defect#	49(34–116)	884(148–2203)	< 0.001
Filling defects location:			0.092
Pulmonary artery stump	33(72%)	28(90%)	
Pulmonary vein stump	13(28%)	3(10%)	
Stump length* (cm)	1.33 ± 081	1.60 ± 0.89	0.145
Filling defect long axis length* (cm)	1.06 ± 0.53	1.47 ± 0.85	0.010
Attenuation* (Hounsfield unit)	36 ± 7	42 ± 10	0.405
Findings at follow-up (all):			0.001
Increased	7(15%)	16(52%)	
Stabilization and/or regression	39(85%)	15(48%)	
Findings at follow-up(Non-anticoagulant):			0.001
Increased	3(9%)	15(52%)	
Stabilization and/or regression	29(91%)	14(48%)	
Findings at follow-up (Anticoagulant):			1.000
Increased	4(29%)	1(50%)	
Stabilization and/or regression	10(71%)	1(50%)	
Time to complete resolution#	223(108–456)	204(19–992)	0.694

#: days, median (ranges)

*: mean ± standard deviation

### Influence of anticoagulant treatment

Anticoagulants used in the treatment included low-molecular-weight heparin, Warfarin, or Rivaroxaban. A total of 16 patients received anticoagulant therapy, 11/16 (68.8%) filling defects showed stability or regression. In the no anticoagulant group (*n* = 61), 43/61 (70.4%) filling defects showed stability or regression. There were no significant differences in the influence on filling defects between the anticoagulant and non-anticoagulant groups (*P* = 1.000)(Table 3). The anticoagulant group had a shorter complete resolution time (median: 187 days, IQR: 60–423 days) compared to the non-anticoagulant group (median: 343 days, IQR: 152–767 days), but the difference was not statistically significant (*P* = 0.284).

**Table 3:** Impact of anticoagulant treatment on pulmonary vascular stump filling defects post lung cancer resection

	Anticoagulant (*N* = 16)	Non-anticoagulant (*N* = 61)	*P*
Findings at follow-up (all):			*P* = 1.000
Increased	5(31%)	18(30%)	
Stabilization and/or regression	11(69%)	43(70%)	
Findings at follow-up (Convex):			*P* = 0.168
Increased	4(29%)	3(9%)	
Stabilization and/or regression	10(71%)	29(91%)	
Findings at follow-up (Concave):			*P* = 1.000
Increased	1(50%)	15(52%)	
Stabilization and/or regression	1(50%)	14(48%)	
Complete resolution (all):			*P* = 1.000
yes	7(44%)	26(43%)	
no	9(56%)	35(57%)	
Time to complete resolution#	187(60–423)	343(152–767)	*P* = 0.284

#: days, median (ranges)

Among the 16 patients who received anticoagulant therapy, two patients experienced gastrointestinal bleeding, and one patient had cerebral haemorrhage. It is worth noting that in five cases, the filling defects gradually increased despite ongoing the anticoagulation treatment, with three cases showing tumor recurrence and two cases demonstrating metastasis. Among these five patients, following the discontinuation of anticoagulation therapy and continuing with regular anticancer treatment, on their latest CT scan, one filling defect had completely resolved, two cases had partially resolved, and one case remained stable.

During a median follow-up period of 371 days (IQR: 182–704 days), with a range from 28 to 2223 days, no filling defect-related complications were observed. On the final follow-up CT scan, among the 23 cases of increased filling defects during short-term follow-ups, 11 filling defects remained unchanged or decreased, and none of these eight filling defects received the anticoagulant treatment.

### PFS analysis of filling defects

The median follow-up time for all patients was 371 days (IQR, 182–704 days), with a range from 28 to 2223 days. In the case group, a total of 30 patients reached the endpoint event during follow-up, while 47 patients did not experience an endpoint event during follow-up. 28 experienced tumor progression, and 2 deaths occurred due to tumor progression. 12 patients with convex-shaped filling defects and 18 patients with concave-shaped filling defects experienced endpoint events, respectively.

**Fig. 3:**
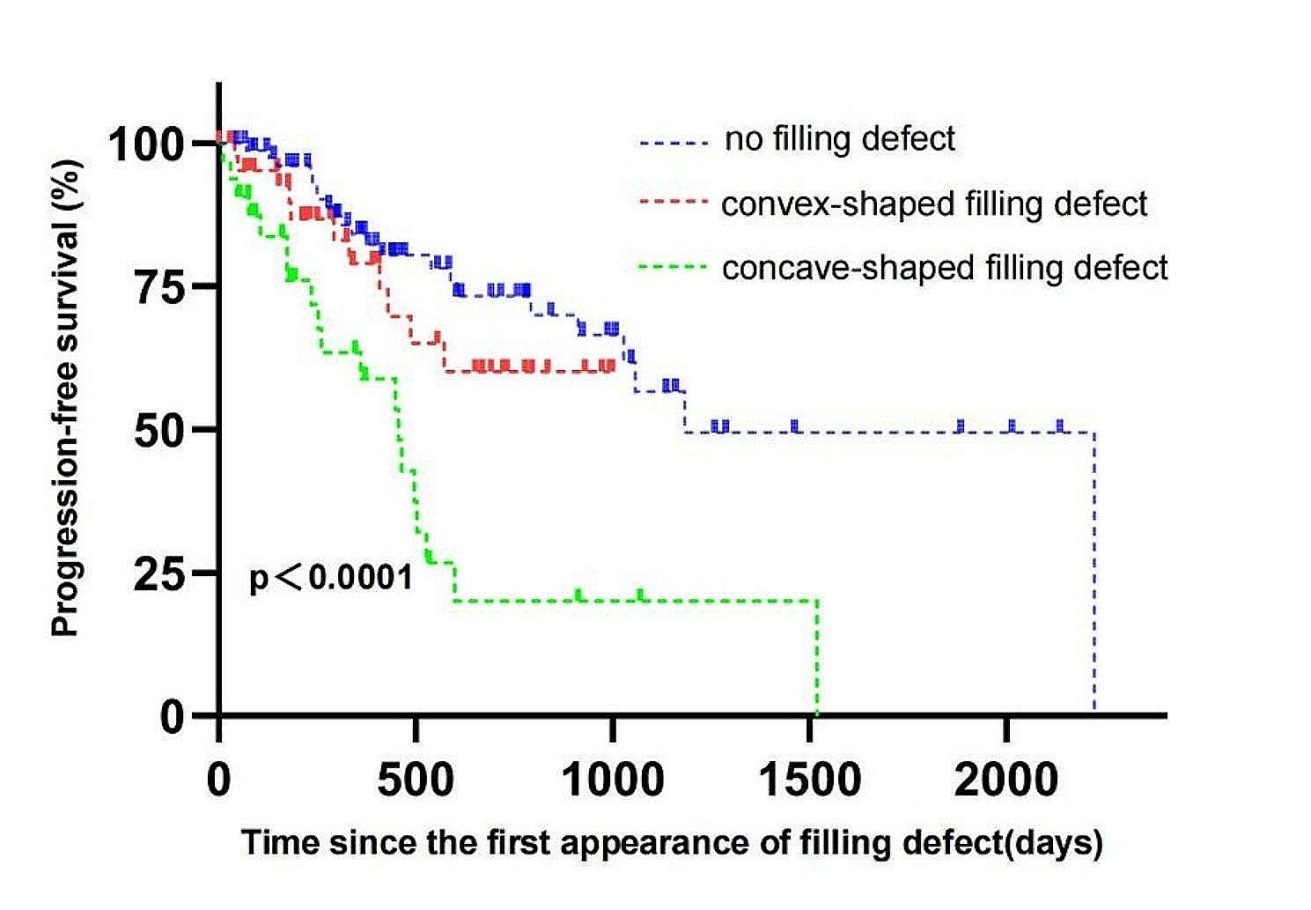
Kaplan–Meier subgroup progression-free survival analysis. There was a statistically significant difference in the progression-free survival rate between the concave-shaped filling defects and the no filling defects groups (log-rank *P* < 0.0001), as well as between the concave-shaped filling defects and convex-shaped filling defects groups (log-rank *P* < 0.0085). However, no statistically significant difference in progression-free survival was observed between the convex filling defects group and the no filling defects group (log-rank *P* = 0.2807)

Kaplan-Meier survival analysis revealed a statistically significant difference in PFS among the groups of concave-shaped filling defects, convex-shaped filling defects, and no filling defects (log-rank *P* < 0.0001) (Fig. 3). The median survival time without progression was the shortest in the concave-shaped filling-defect group, at 456 days (95% CI: 425–486 days), the longest in the no filling-defect group, with an average of 1439 days (95% CI: 1159–1718 days), and the median in the convex-shaped filling-defect group, with an average of 715 days (95% CI: 586–844 days). After pairwise comparison, there was a statistically significant difference in the PFS rate between the concave-shaped filling-defect and no filling-defect groups, as well as between the concave-shaped filling-defect and convex-shaped filling-defect groups (log-rank *P* < 0.0001, log-rank *P* < 0.0085). However, there was no statistically significant difference in PFS between the convex filling-defect group and the no filling-defect group (log-rank *P* = 0.2807).

In the univariate Cox proportional hazards analysis of the filling-defect subgroups (Table 4), shape of filling defect (HR 0.43, 95% CI 0.21–0.91; *P* = 0.026), preoperative staging of cancer (HR 2, 95% CI 0.96–4.17; *P* = 0.07), and development of filling defects (HR 5.8, 95% CI 2.67–12.62; *P* < 0.001) adversely affected PFS. However, gender (*P* = 0.137), age (*P* = 0.692), histopathological type (*P* = 0.16), surgical intervention (*P* = 0.439), thrombosis location (*P* = 0.20), and attenuation (Hounsfield unit) (*P* = 0.295) did not significantly impact PFS. The histopathological type, despite having a *P*-value greater than 0.10 in the univariate analysis, was included in the following analysis due to its clinical relevance.

**Table 4:** Cox proportional hazard regression of risk factors for tumor-progression with pulmonary-vascular-stump filling defect

	Univariable Cox proportional hazard regression		Multivariable Cox proportional hazard regression(early onset)		Multivariable Cox proportional hazard regression(follow-ups)	
	HR (95% CI)	*P*	HR (95% CI)	*P*	HR (95% CI)	*P*
Gender:						
Male	Reference					
Female	1.92 (0.85–4.36)	0.137				
Age, years:						
≤ 64	Reference					
>64	0.86 (0.41–1.8)	0.692				
Histological types:						
Non-small cell	Reference		Reference		Reference	
Small cell	1.3(0.41–4.54)	0.610	0.71(0.42–0.83)	0.400	1.27(0.38–4.30)	0.696
Stage of cancer at surgery:						
Stage I/II	Reference		Reference		Reference	
Stage III/IV	2 (0.96–4.17)	0.070	1.18(0.38–1.55)	0.120	0.81(0.38–1.75)	0.592
Surgical intervention:						
Right side	Reference					
Left side	0.75 (0.36–1.56)	0.439				
Location:						
Pulmonary artery stump	Reference					
Pulmonary vein stump	0.50 (0.17–1.43)	0.200				
Shape:						
Convex shape	Reference		Reference		Reference	
Concave shape	0.43 (0.21–0.91)	0.026	0.46(0.39–1.99)	0.040	0.46(0.39–1.99)	0.757
Attenuation(Hounsfield unit)						
≤ 38	Reference					
>38	1.52 (0.69–3.35)	0.295				
Findings at follow-up:						
Stabilization or regression	Reference					
Increased	5.8 (2.67–12.62)	< 0.001			0.26(0.11–0.65)	0.004

In the multivariate Cox proportional hazards analysis showed that the concave shape of filling defects was found to be an independent predictor of PFS in early onset of the pulmonary vascular stump filling defect on CT, with an HR of 0.46 (95% CI 0.39–1.99; *P* = 0.04) (Table 4). While in the follow-ups of the pulmonary vascular stump filling defect on CT, the independent predictor of PFS was the increase in the size of filling defects (HR 0.26 95% CI 0.11–0.65; *P* = 0.004) (Table 4).

Finally, radiation, chemotherapy, stage III-IV staging, histologic type, or positive stump pathology at resection patients who developed concave filling defects, divided into subgroups of “ increased concave filling defects” and “stability or regression concave filling defects”, are presented in Table 5.

**Table 5:** Analysis of clinical characteristics of concave filling defect.Histological type

	Concave filling-defects(*N* = 15) Increased	Concave filling-defects(*N* = 14) stability or regression	*P*
Chemotherapy	7(46.7%)	6(42.8%)	1.000
Radiotherapy	5(33.3%)	4(28.6%)	1.000
Stage of cancer at resection:			0.700
Stage I/II	9(60.0%)	10(71.5%)	
Stage III/IV	6(40.0%)	4(28.5%)	
Histological type:			0.715
Adenocarcinoma	9(60.0%)	7(50.0%)	
Non-adenocarcinoma	6(40.0%)	7(50.0%)	
Positive stump pathology	5(33.3%)	2(14.2%)	0.390

## Discussion

In previous studies, filling defects in the vascular stump after lung cancer resection had been considered as bland thrombus. In our study a minority of filling defects in the arterial stump (6.5%) showed the nuclide concentration on PET, developing into extravascular extension from the intra-artery filling defect during CT follow-ups. These findings were indicative of arterial stump recurrence (Fig. 2), and the filling defect was suspected to be associated with cancer thrombus implanted at the vascular stump during lung resection, since its invasive propagation and with nuclide concentration on PET. The pulmonary-vascular-stump filling defect not only could represent an in-situ thrombosis or an embolus from deep vein thrombosis, but also might indicate a cancer recurrence inside the vascular stump. To the best of our knowledge, pulmonary-vascular-stump recurrence has never been reported before.

Since the majority of the pulmonary-vascular-stump filling defects were thrombi, previous studies primarily focused on investigating the prognosis of thrombi themselves. Corresponding research results indicate an extremely low probability of secondary pulmonary embolism and conclude the benign natural history of thrombus [1, 5, 6]. However, the relationship between the appearance of thrombi, indicative of a hypercoagulable state, and the progression of tumors, was neglected. In fact, cancer cells can directly activate platelets and the coagulation system within the bloodstream or indirectly through the production of microparticles and/or secreted factors and cytokines. Consequently, activated platelets actively contribute to tumor progression and the formation of metastasis [9, 10]. Some coagulation factors, aside from their critical role in blood clotting, have also demonstrated involvement in cancer development and progression [11].

We conducted a PFS analysis of patients with postoperative pulmonary-vascular-stump filling defects. Patients with concave-shaped filling defects had the shortest PFS, while patients without filling defects showed the longest PFS, with a statistically significant difference. Multivariable Cox proportional hazards analysis showed that the influencing factor for PFS in early onset was the shape of filling defects. Previous studies revealed that a higher neutrophil and red blood cell content in convex-shaped thrombi is primarily linked to injury of endothelial cells, while the formation of dense fibrous material in concave-shaped thrombi is mainly associated with hypercoagulability [12]. This finding aligns with our conclusion that concave-shaped filling defects are correlated with tumor recurrence or progression, as concave-shaped filling defects are associated with the hypercoagulable state potentially induced by tumor activity.

Subsequently, we conducted multivariable Cox proportional hazards analysis during the follow-ups, which revealed that the increase in the size of filling defect was an independent factor influencing PFS upon follow-ups. Patients with increased filling defects during short-term follow-ups, especially those with increased filling defects despite anticoagulant therapy, had a shorter PFS. One interpretation of this phenomenon is that the filling defect represents a cancer thrombus. The expansive and irregular growth observed could be indicative of the dynamic nature of a cancer thrombus. Another plausible explanation is that the filling defect corresponds to a blood clot. The clot’s growth may reflect the persistent hypercoagulation status in patients with lung cancer, suggesting ongoing activity and progression of tumor cells in the bloodstream even after tumor resection. Therefore, the growth of the filling defect overwhelmed its morphological characteristics in predicting tumor prognosis during follow-ups.

Even though these filling defects were considered clots, there has always been significant controversy regarding the necessity of anticoagulant therapy [13–15]. In our study, there was no statistically significant difference in the regression rates of filling defects between the anticoagulant group and the non-anticoagulant group, which is consistent with previous studies [4, 6]. While, out of 16 cases receiving anticoagulant therapy, adverse reactions associated with anticoagulant therapy included two cases of gastrointestinal bleeding, one case of cerebral hemorrhage. This showed that anticoagulant therapy did not significantly affect filling-defect resolution but potentially increased the risk of bleeding. However, anticoagulant medications promise to play a role in minimizing the tumor-promoting defects, not only by inhibiting direct products, but also by inhibiting tissue penetration, angiogenesis, and local stimulation of trophic factors. The benefits of low-molecular-weight heparins in the evolution of lung cancer was revealed early [16]. Therefore, it is advisable to exercise caution in the use of anticoagulant therapy, carefully considering the balance between potential benefits and risks.

To the best of our knowledge, this study is the inaugural exploration delving into the promising malignant nature of pulmonary-vascular-stump filling defects following lung cancer resection. Additionally, it stands as the most extensive series investigating the prognosis of lung cancer patients with these filling defects. However, several limitations warrant acknowledgment. Firstly, the absence of pathological confirmation arises from the practical challenges of obtaining such results in a clinical context. Secondly, the inherent variability in follow-up time and frequency, characteristic of postoperative scenarios, has led to some data loss, potentially affecting the robustness of our findings. Lastly, not all subjects underwent PET imaging.

## Conclusions

On conclusion, our study revealed malignant growth in certain pulmonary-arterial-stump filling defects following lung cancer resection. In the early onset of filling defects on CT, the concave shape of filling defects emerged as an independent predictor of cancer progression, while during the subsequent follow-up, an increase in the size of filling defects can independently predict cancer progression.

## Data Availability

The datasets used and/or analysed during the current study are available from the corresponding author on reasonable request.

## References

[1] Kwek BH, Wittram C (2005). Postpneumonectomy pulmonary artery stump thrombosis: CT features and imaging follow-up. Radiology.

[2] Kim SY, Seo JB, Chae EJ, Do KH, Lee JS, Song JW (2005). Filling defect in a pulmonary arterial stump on CT after pneumonectomy:radiologic and clinical significance. Am J Roentgenol.

[3] López-Padilla D, Peghini Gavilanes E, Revilla Ostolaza TY, Trujillo MD, Martínez Serna I, Arenas Valls N (2016). Arterial stump thrombosis after lung resection surgery: clinical presentation, treatment and progress. Arch Bronconeumol.

[4] Gurel Durmus Z, Bulbul Y, Tekinbas C, Seyis KN, Kosucu P (2022). Frequency and predictors of pulmonary arterial stump thrombosis following pneumonectomy or lobectomy. Med Princ Pract.

[5] Moon MH, Beck KS, Moon YK, Park JK, Sung SW (2017). Incidence and clinical features of the incidentally found vascular stump thrombus during routine follow up after oncologic lung surgery. PLoS ONE.

[6] Park JE, Cha SI, Lee DH, Lee EB, Choi SH, Lee YH (2022). Pulmonary vein stump thrombosis after lung resection for lung cancer: clinical features and outcome. Blood Coagul Fibrinolysis.

[7] Yamamoto T, Suzuki H, Sakairi Y, Iwata T, Iizasa T, Tagawa T, Yoshida S, Takemura R, Sato Y, Yoshino I (2023). Thrombus formation at the stump of the pulmonary vein after lobectomy: a prospective multi-institutional study. Surg Today.

[8] Trousseau A. Phlegmatia alba dolens. In Clinique Médicale de L’hôtel-dieu de Paris, 2nd ed.; J.-B. Baillière et fils: Paris, France, 1865;3:654–712.

[9] Falanga A, Marchetti M, Vignoli A (2013). Coagulation and cancer: biological and clinical aspects. J Thromb Haemost.

[10] Mege D, Panicot-Dubois L, Dubois C (2019). Mechanisms of cancer-associated thrombosis. Hemasphere.

[11] Ahmadi SE, Shabannezhad A, Kahrizi A, Akbar A, Safdari SM, Hoseinnezhad T, Zahedi M, Sadeghi S, Mojarrad MG, Safa M (2023). Tissue factor (coagulation factor III): a potential double-edge molecule to be targeted and re-targeted toward cancer. Biomark Res.

[12] Hashimoto H, Usui G, Tsugeno Y, Sugita K, Amori G, Morikawa T, Inamura K (2019). Cerebral thromboembolism after Lobectomy for Lung Cancer: pathological diagnosis and mechanism of Thrombus formation. Cancers (Basel).

[13] Mirijello A, Santoliquido M, Piscitelli P, Borelli C, Serviddio G, Simeone A (2021). Pulmonary artery stump thrombosis: to treat or not to treat? The question is still open. Description of a case and review of the literature. Front Cardiovasc Med.

[14] Wieteska-Miłek M, Winiarczyk K, Kupis W (2021). Treatment of pulmonary artery stump thrombosis after lobectomy: a case report and literature review. Adv Respir Med.

[15] Chaaya G, Vishnubhotla P (2017). Pulmonary vein thrombosis: a recent systematic review. Cureus.

[16] Altinbas M, Coskun HS, Er O, Ozkan M, Eser B, Unal A (2004). A randomized clinical trial of combination chemotherapy with and without low-molecular-weight heparin in small cell lung cancer. J Thromb Haemost.

